# Animal models for photodynamic therapy (PDT)

**DOI:** 10.1042/BSR20150188

**Published:** 2015-11-13

**Authors:** Zenildo Santos Silva, Sandra Kalil Bussadori, Kristianne Porta Santos Fernandes, Ying-Ying Huang, Michael R. Hamblin

**Affiliations:** * Massachusetts General Hospital, Wellman Center for Photomedicine, Boston, MA 02114, U.S.A.; † Harvard Medical School, Department of Dermatology, Boston, MA 02114, U.S.A.; ‡ Postgraduate Program in Biophotonics Applied to Health Sciences, Nove de Julho University, São Paulo, SP 02117-010, Brazil; § Harvard-MIT Division of Health Sciences and Technology, Cambridge, MA 02139, U.S.A.

**Keywords:** autochthonous, cancer, genetically-engineered mouse model, infection, orthotopic tumour, photodynamic therapy, subcutaneous tumour, xenograft

## Abstract

Photodynamic therapy (PDT) employs non-toxic dyes called photosensitizers (PSs), which absorb visible light to give the excited singlet state, followed by the long-lived triplet state that can undergo photochemistry. In the presence of ambient oxygen, reactive oxygen species (ROS), such as singlet oxygen and hydroxyl radicals are formed that are able to kill cancer cells, inactivate microbial pathogens and destroy unwanted tissue. Although there are already several clinically approved PSs for various disease indications, many studies around the world are using animal models to investigate the further utility of PDT. The present review will cover the main groups of animal models that have been described in the literature. Cancer comprises the single biggest group of models including syngeneic mouse/rat tumours that can either be subcutaneous or orthotopic and allow the study of anti-tumour immune response; human tumours that need to be implanted in immunosuppressed hosts; carcinogen-induced tumours; and mice that have been genetically engineered to develop cancer (often by pathways similar to those in patients). Infections are the second biggest class of animal models and the anatomical sites include wounds, burns, oral cavity, ears, eyes, nose etc. Responsible pathogens can include Gram-positive and Gram-negative bacteria, fungi, viruses and parasites. A smaller and diverse group of miscellaneous animal models have been reported that allow PDT to be tested in ophthalmology, atherosclerosis, atrial fibrillation, dermatology and wound healing. Successful studies using animal models of PDT are blazing the trail for tomorrow's clinical approvals.

## Abstract (precis)

Photodynamic therapy (PDT) combines visible light and photosensitizing dyes. Different animal models have been used to test PDT for cancer, infectious disease and cardiovascular disease. Mouse models of tumours include subcutaneous, orthotopic, syngeneic, xenograft, autochthonous and genetically modified.

## INTRODUCTION

Photodynamic therapy (PDT) employs non-toxic dyes called photosensitizers (PSs), which absorb visible light of the correct wavelength to first give the excited singlet state, followed by a transition to the long-lived excited triplet state that can undergo photochemistry [1]. In the presence of molecular oxygen, the photochemical reactions produce a variety of reactive oxygen species (ROS) including singlet oxygen and hydroxyl radicals. These ROS cause oxidative damage to proteins, lipids and nucleic acids and leading to cell death by necrosis and/or apoptosis (Figure 1), PDT was originally developed as a cancer treatment, but has subsequently been investigated as a treatment for choroidal neovascularization secondary to age-related macular degeneration, for a range of localized infections and for disorders related to dermatology and immunology.

**Figure 1:**
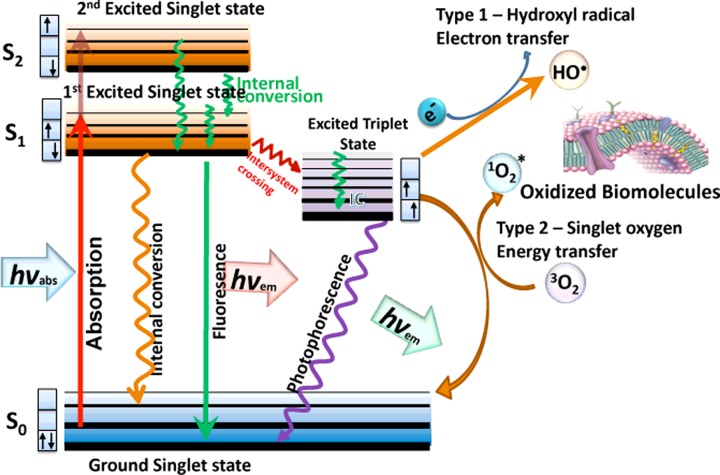
Jablonski diagram When light (hv) is absorbed by the PS the electron moves from a non-excited, low-energy singlet state into a high-energy singlet state. This excited state can lose energy by emitting a photon (fluorescence) or by internal conversion (non-radiative decay). The process known as inter-system crossing, involves flipping of the spin of the high-energy electron, leading to a long-lived excited triplet state. In the presence of molecular oxygen, superoxide and hydroxyl radicals are formed in type I reactions and singlet oxygen in type II reactions. These ROS can damage most types of biomolecules (proteins, lipids, nucleic acids).

When PDT is used as a tumour therapy, the PS is usually injected IV (intravenous), followed by a waiting period known as the ‘drug light interval’ to allow the tumour to accumulate at the site of the tumour. Light can be delivered to the tumour site either by shining a focused spot of light on to the tissue (which can be done endoscopically) or by inserting fibre optics into the tumour tissue in a technique called interstitial light delivery. The anti-tumour effects of PDT result from the combination of three different *in vivo* mechanisms, namely direct PDT cytotoxicity to tumour cells, destruction of the tumour microvasculature and induction of an acute local inflammatory response leading to activation of the host immune system (Figure 2).

**Figure 2:**
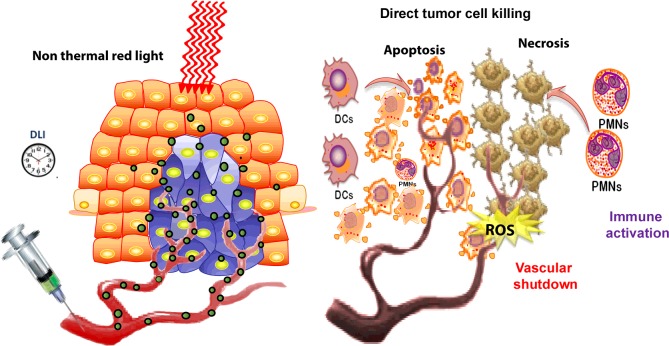
Mechanisms of PDT of an experimental tumour PS is injected IV followed by a time delay [drug-light interval (DLI)]. Then activating red light is delivered to the tumour cause the production of ROS. The ROS can cause direct tumour cell killing by necrosis or apoptosis, shut-down the tumour blood supply and activate neutrophils (PMN) and dendritic cells (DC) that stimulates an anti-tumour immune response.

Many different types of photoactivable molecules have been synthesized and tested as possible PDT agents. These PS are often based on the tetrapyrrole backbone such as porphyrins, chlorins, bacteriochlorins and phthalocyanines (Figure 3). The latter three structures possess strong light absorption bands at wavelengths longer than 650 nm and are therefore well suited to the so-called ‘optical window’ required for good tissue penetration of light. Highly effective PS require an absorption maximum between 650 and 800 nm to avoid the absorption by the endogenous tissue chromophores, such as haemoglobin, whereas still having enough photon energy to carry out photochemistry. The chemical structure of the PS molecule can be tailored to provide high cell uptake, selectivity for cancer cells and endothelial cells and to provide photostability (i.e. resistance to photobleaching). A recent alternative approach is to attach the PS covalently or non-covalently to biomolecules that possess a marked targeting ability towards cancer cells, such as monoclonal antibodies or specific peptides. A popular alternative to traditional PS is to use 5-aminolevulinic acid (ALA), a biochemical precursor to the endogenous PS, protoporphyrin IX (PPIX) [2].

**Figure 3:**
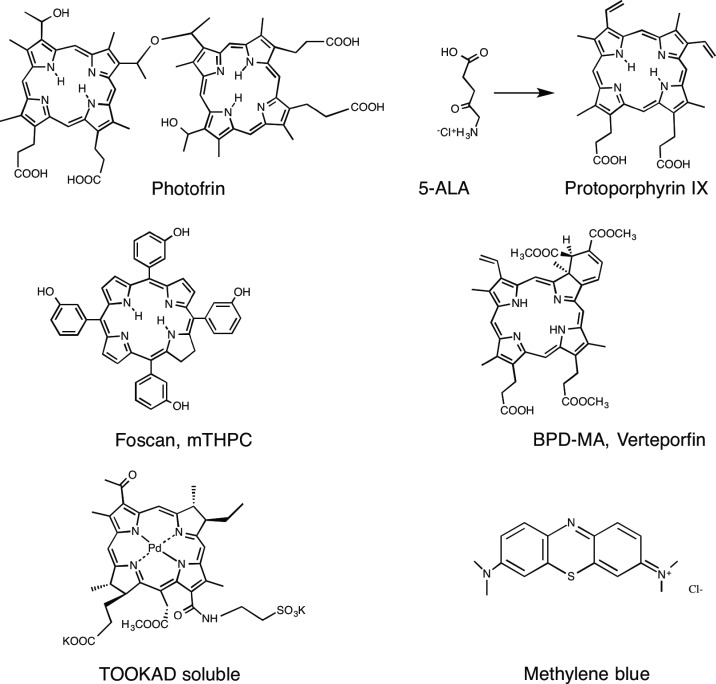
Chemical structures of some representative PS that have been applied in both pre-clinical animal models and also in clinical studies Shown are Photofrin, conversion of ALA to PPIX, Foscan or mTHPC (m-tetrahydroxyphenylchlorin), Verteporfin or BPD-MA (benzoporphyrin derivative monoacid ring A), TOOKAD (palladium bacteriopheophorbide) soluble and methylene blue.

### Cancer

Since the first pioneering studies of PDT to cure tumours in the 1970s by Diamond et al. [3] and by Dougherty et al. [4], cancer has been the leading indication for PDT. Although much research has been carried out in cell culture studies *in vitro* and, more recently, in 3D tissue culture models [5,6], more complex systems such as laboratory animals are required to demonstrate that these new PDT approaches could eventually work in clinical situations. The next sections of this review will give an overview of the different animal models that have been employed in studies of PDT for cancer.

### Chorioallantoic membrane

One very simple intermediate model that lies in between *in vitro* cell culture and laboratory animals is the chorioallantoic membrane (CAM) of fertilized chicken eggs that have had a window of eggshell removed. This model allows the growth of tumour cells that are applied as a suspension on to the surface of the membrane and turn into ‘tumours’ that go on to develop their own blood supply by the process of angiogenesis (in a similar fashion to real tumours in mice). PS can be injected into these blood vessels, allowed to accumulate in the tumours and then light can easily be delivered and changes in blood flow in the tumour and normal vessels can be observed in real time. PS can also be topically applied to the xenografted tumours on the CAM. The advantages of this model include the ease of *in vivo* microscopy to study PDT-induced vascular damage and the lack of regulatory controls on experiments involving eggs [7–11].

### Subcutaneous syngeneic mouse and rat tumours

Using subcutaneous tumours in laboratory mice is very common among investigators aiming to test various PDT regimens (Table 1). There are a large number of mouse tumour cell lines available, which differ in the type of tissue or organ that the tumour originated from and also in the particular syngeneic inbred mouse strain that the tumours belong to. Individual laboratory mouse strains (*Mus musculus*) have particular combinations of major histocompatibility complexes (MHCs; MHC class I such as H2B and MHC class II such as Ia) and the tumour cells should have the same combination of MHC molecules as the host mouse to allow them to grow without instant rejection. This syngeneic mouse tumour approach is often used because the mice have intact immune systems and, therefore, immunology and anti-tumour immunity effects after PDT can be studied. There are many inbred mouse strains available (over 400), but the most commonly used examples in anti-cancer PDT studies are BALB/c (albino), C57BL/6 (black), C3H (brown) and DBA2 (grey). Tumour types include adenocarcinoma lines (colon, lung, breast, pancreas etc.), squamous cell carcinoma, fibrosarcoma, lymphoma, melanoma, malignant glioma and many others. Rat tumour cell lines are less common than murine tumour lines, but there are several known belonging to inbred laboratory rat strains (*Rattus norwegicus*) such as Wistar, Lewis, Sprague–Dawley, Fischer 344 etc. Subcutaneous tumours differ in their rate of growth, degree of vascularization, amount and kind of tumour stroma and potential to form spontaneous metastasis. In addition to injecting the tumour cells beneath the skin, they can also be injected between the layers of the epidermis and dermis to form intradermal tumours. It has been shown that intradermal tumours are more easily recognized by the immune system compared with subcutaneous tumours [12]. In some cases, investigators mix the tumour cells with a preparation of extracellular matrix, called Matrigel, before injection and it has been shown that this can make a difference in the response of the tumour to PDT [13]. Many investigators transplant tumours from one animal to another by inserting small pieces obtained from an excised whole tumour under the skin of the next animal.

**Table 1:** Subcutaneous syngeneic mouse/rat tumours that have been used for PDT Abbreviations: AIS2Pc, aluminum phthalocyanine disulfonated (adjacent); AO, acridine orange; BPD-MA, benzoporphyrin derivative monoacid ring A; DLI, drug light interval; HPPH, hexyl-pyropheophorbide; inj, injected; mTHPC, m-tetrahydroxyphenylchlorin; TPP, tetraphenylporphyrin.

Cell line	Cancer type	Mouse strain	PDT regimen	References
EMT6	Mammary sarcoma	BALB/c	Photofrin, DLI 24 h, 630 nm, 110 J/cm^2^, 130 mW/cm^2^	[38]
CT26 (c26, colo26)	Colon adenocarcinoma	BALB/c	Pheophorbide HPPH, DLI 24 h, 665 nm, 100 J/cm^2^, 75 mW/cm^2^	[39]
DA3^Hi^	Breast adenocarcinoma	BALB/c male mice	Hypericin, inj IP, DLI 6 h, 400–700 nm, 60 J/cm^2^, 20 min	[40]
MS-2	Fibrosarcoma	BALB/c	Phthalocyanine AIS2Pc, DLI 24 h, 670 nm, 60 J/cm^2^. 100 mW/cm^2^	[41]
4T1	Breast cancer	BALB/c female mice	Verteporfin BPD-MA, DLI 24 h, 690 nm, 120 J/cm^2^	[42]
B16	Melanoma	C57BL/6	Porphyrin TPP, DLI 9 h, 630 nm, 90 J/cm^2^, 100 mW/cm^2^,	[43]
LLC	Lewis lung adenocarcinoma	C57BL/6	Photofrin, DLI 24 h, 630 nm, 150 J/cm^2^, 90 mW/cm^2^	[44]
TC1	Lymphoma	C57BL/6	Radachlorin, DLI 3 h, 662 nm, 300 J/cm^2^,	[45]
RIF1	Radiation-induced fibrosarcoma	C3H	Photofrin, DLI 24 h, 400 nm, 135 J/cm^2,^	[46]
FsaR	Fibrosarcoma	C3H/HeN	Photofrin, DLI 24 h, 630 nm, 150 J/cm^2^, 110 mW/cm^2^	[47]
SCCVII	Squamous cell carcinoma	C3H	Foscan mTHPC, DLI 24 h, 650 nm, 50 J/cm^2^, 90 mW/cm^2^	[48]
DLM-8	Osteosarcoma	C3H	Acridine orange AO, DLI 2 h, xenon flash lamp, 15 J per pulse, 60 Hz, 10 min	[49]
P815	Mastocytoma	DBA/2	Verteporfin BPD-MA, DLI 15 min, 690 nm, 120 J/cm^2^, 100 mW/cm^2^	[50]
Sarcoma 180	Sarcoma	ICR outbred	Haematoporphyrin, inj IP, DLI 24 h, 635 nm, 30 J/cm^2^, 26 mW/cm^2^	[51]
NXS2	Neuroblastoma	Female A/J	Pheophorbide HPPH, DLI 24 h, 665 nm, 48 J/cm^2^, 7 mW/cm^2^	[52]
VMDK	Glioma	VM	Porphyrin mTHPP, DLI 24 h, 648 nm, 20 J/cm^2^, 300 mW/cm^2^.	[53]
Walker 256	Carcinosarcoma	Wistar male rats	Porphyrin TSPP, DLI 24 h, 685 nm, 50 J/cm^2^, 25 W/cm^2^	[54]
MatLyLu (Dunning)	Prostate	Copenhagen rats	Verteporfin BPD, DLI 1 h, 690 nm, 50 J/cm^2^, 50 mW/cm^2^	[55]

PDT of subcutaneous mouse tumours is usually carried out by IV injection of the PS (often in the tail vein) followed after a certain period of time by light delivery to the tumour including a certain amount of surrounding normal tissue. This time period, called the ‘drug light interval’, is an important parameter that governs the amount of vascular damage that occurs during the treatment and therefore whether the PDT regimen is ‘vascular’ or ‘cellular’. Less common is the injection of the PS into the abdominal cavity (intraperitoneal [14] or directly into the tumour (intra-tumoural [15]). With the recent rise in the study of PDT mediated by a wide range of nanoparticles and multifunctional nanostructures, the use of intra-tumoural injection has become more common. This is because the pharmacokinetics and bio-distribution of these engineered nanostructures has as yet not been much investigated.

The response of subcutaneous tumours depends on whether the PDT regimen is predominantly vascular or cellular. For vascular PDT, a circumscribed black eschar usually appears approximately 2 days post PDT (Figure 4). For cellular PDT, it is common for the tumour volume to shrink without such a visible black eschar. In many cases, a pronounced swelling of the tissue is seen soon after completion of the illumination, due to the acute inflammatory reaction. The duration of the tumour response depends on many factors. In the case of a marked immune response occurring after PDT, tumours do not recur and the animals are regarded as cured after 90 days of being tumour-free. Local tumour recurrence can occur after PDT and this recurrence often takes the form of an annulus or ‘doughnut’ of tumour surrounding the original tumour. The reason for the morphology of this recurrence is not completely understood, but may involve the presence of cancer stem cells in the periphery of the original tumour at the ‘invasive front’ [16].

**Figure 4:**
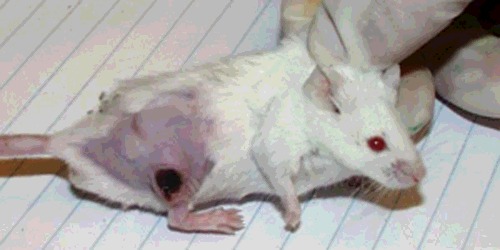
Image of a subcutaneous mouse tumour (EMT6 breast carcinoma in BALB/c mouse) treated with PDT (iv BPD 1 mg/kg, 15 min drug-light interval, 100 J/cm^2^ 690 nm light) Three days post-PDT, a circumscribed black eschar is visible that will heal with no detectable scar after 2 weeks.

### Human xenograft tumours

Much more is known about the molecular biology and tumour biology of human tumours than is known for analogous mouse tumours. A large amount of knowledge about mutations, oncogenes, impaired tumour suppressor pathways, growth factors, epigenetics and so on, is more or less specific for human tumours and much less is known about the effects of these factors in mouse tumours. The discovery of immunosuppressed mice that can be either athymic nude mice or the severe combined immunodeficient (SCID) mice that are T- and B-cell-deficient, allows the engraftment of human tumour cells known as ‘xenografts’ (or sometimes xenographs). Due to the increased susceptibility of these mice to infections, the cell lines must be free of mouse pathogens and the mice must be kept in specific pathogen-free conditions. Even so, innate immunity, particularly natural killer (NK) cells and tumouricidal macrophages, can limit tumour growth and prevent metastasis in nude mice [17]. A large number of human cancer cell lines grown as xenograft tumours grown in a subcutaneous location in either nude mice or SCID mice have been subjected to PDT (Table 2).

**Table 2:** Orthotopic syngeneic mouse/rat tumours Abbreviations: DLI, drug light interval; EGFpep–AuNP-Pc4, a conjugate between an epidermal growth factor peptide, gold nanoparticles, and silicon phthalocyanine; mTHPC, m-tetrahydroxyphenylchlorin.

Cancer type	Cell line	Mouse/rat model	PDT regimen	References
Prostate	TRAMP-C2	Albino male C57BL/6 mice	5-ALA, DLI 72 h, 635 nm, 100 J/cm^2^, 200 mW/cm^2^	[56]
Bladder	AY-27 cells	Female Fischer F344 rats	ALA, DLI 2 h, 514 nm, 20 J/cm^2^, 100 mW/cm^2^	[57]
Prostate	R3327-MatLyLu Dunning	Male Copenhagen rats	Verteporfin DLI 15 min–3 h, 690 nm, 50 J/cm^2^, 50 mW/cm^2^, 1000 s	[58]
Glioma/brain	9L.E29	Female athymic mice	Phthalocyanine, EGF_pep_-Au NP-Pc 4; DLI 4 h, 672 nm, 50 J/cm^2^, 0.1 W/cm^2^	[59]
Breast cancer	4T1	BALB/c female mice	Photofrin, DLI 120 h, 635 nm, 100 J/cm^2^, 416.7 mW/cm^2^	[60]
Glioma/brain	C6-9	Sprague–Dawley male rats	Foscan, mTHPC DLI 48 h, 652 nm, 20 J/cm^2^, 100 mW/cm^2^	[61]
Glioma/brain	BT4C	BD-IX rats	ALA (ip), 5 h DLI, 632 nm, 26 J, 30 mW	[62]

### Orthotopic syngeneic mouse and rat tumours

There has been criticism levelled at the widespread use of subcutaneous tumours in PDT research, to the effect that they fail to replicate the normal biology of human tumours by not being situated in their organ of origin (orthotopic) and are therefore not subject to the appropriate environmental cues and signalling from the host tissue. The blood supply that develops to supply the tumour is very different in orthotopic models and the propensity to spontaneously metastasize is also higher. For instance brain tumours behave very differently when implanted in the brain, than when grown subcutaneously. Investigators have therefore injected the tumour cells into the tissue of the organ of origin in the mouse or rat. This procedure may need a surgical approach that depends on the actual organ to be implanted with the tumour. This has been done with colon carcinomas (into the wall of the colon), renal cell cancers (into the kidney), mammary carcinomas (into the mammary fat pad), bladder carcinomas (into the bladder wall), prostate carcinoma (into the prostate), pancreatic carcinoma (into the pancreas) and lung cancer (into the bronchi). Moreover, the actual PDT treatment approach necessitates being able to get the light to the tumour, which is more difficult for orthotopic tumours.

Orthotopic tumours used to test PDT can either be syngeneic in mice or rats or can be xenografts (Table 3). There is one rabbit tumour that has been quite often used to create orthotopic tumour models in a medium sized animal, the New Zealand white rabbit. The VX2 tumour cell line was originally derived from a virus-induced skin papilloma [18], but has been widely used to produce tumours in the liver [19], the pancreas [20] and the brain [21], all of which have been treated with PDT.

**Table 3:** Xenograft mouse or rat tumours, Route: IV, IP, IT Abbreviations: AlOH-PC, hydroxyaluminum-phthalocyanine; BPD-MA, benzoporphyrin derivative monoacid ring A; CD1, a strain of mice; DLI, drug light interval; Ncr, another strain of mice; NSCLC, non small cell lung cancer; Pd, TOOKAD, palladium bacteriopheophorbide; sc, subcutaneous; SCC, squamous cell carcinoma.

Cell line	Cancer type	Animal model	PDT regimen	References
OVCAR3	Ovarian	Nude mice	Photofrin, DLI 24 h, 635 nm, 200 J/cm^2^	[63]
OVCAR5	Ovarian	Nude mice	Verteporfin, BPD-MA, DLI 90 min, 690 nm, 40 J, 320 s	[64]
AsPC-1 andPanc-1	Pancreas	Male SCID mice	Verteporfin, DLI 1 h, 690 nm, 40 J/cm^2^, 74mW/cm^2^	[65]
LNCaP	Prostate	SCID mice	Liposomal BPD, DLI 1 h, 690 nm,100 J/cm^2^	[66]
WISH-PC237	Prostate	Male CD1 nude mice	Pd-bacteriopheophorbide TOOKAD, DLI zero, 650–800 nm, 360 J/cm^2^, 30 min	[67]
Eca109	Oesophageal SCC	Nude mice (sc or orthotopic)	Photofrin, DLI 24 h, 630 nm, 135 J/cm^2^, 75 mW/cm^2^	[68]
A549	Lung cancer (NSCLC)	nude mice	Factor VII-targeted Sn(IV) chlorin e6 conjugate, 635 nm, 72 J/cm^2^	[69]
H460	Lung cancer (NSCLC)	Ncr-nu/nu female mice	Photochlor HPPH, DLI 24 h, 661 nm, 200 J/cm. 150 mW/cm	[70]
FaDu	Head and neck SCC	Female nu/nu CByJ.Cg-Foxn1nu/J.	HPPH, DLI 24 h, 665 nm, 48 Jcm^2^, 7 mW/cm^2^,	[52]
MDA-MB 231	Mammary carcinoma	Nude mice	Phthalocyanine, AlOH-PC, DLI 10 min, 635 nm, 100 J/cm^2^, 0.97 W, 7 min.	[71]
MCaIV	Breast	Female SCID	Pyropheophorbide MV6401, DLI 15 min, 664 nm, 5 J/cm^2^, 50 mW/cm^2^.	[72]
HT29	Liver	Female Swiss nude mice	Monoclonal antibody chlorin(e6) conjugate, 17.1A-pl-ce6-succ, DLI 3 h, 666 nm, 80 J, 100 mW, 13.3 min	[73]
NPC	Nasopharyngeal carcinoma	BALB/c nude mice	5-ALA, DLI 3.5 h, 630 nm, 100 J/cm^2^, 100 mW/cm^2^	[74]

### Autochthonous tumours

Tumours that arise naturally in the host are called ‘autochthonous’. In the laboratory studies, these tumours are usually induced by application of chemical carcinogens, but viruses and physical carcinogenic stimuli (for instance UV radiation) have also been used. These models effectively recapitulate the time-dependent and multi-stage progression of cancer formation in response to relevant environmental carcinogens and tumour-promoting agents [22]. These models utilize the topical, intraperitoneal, or oral administration of a variety of polycyclic aromatic hydrocarbons or reactive organic chemicals either alone or in combination with known tumour promoters, such as phorbol esters, to induce specific cancers in a variety of immunocompetent mice, rats and hamsters. The susceptibility to chemical carcinogenesis and the resultant tumour incidence and multiplicity varies with the protocol, the dosage of carcinogens and promoters and the age and strain of the rodents used. The Syrian golden hamster model of oral dysplasia and cancer caused by repeated application of 7,12-dimethyl-benz(a)anthracene (DMBA) is particularly noteworthy, as hamsters are chosen because of the presence of the cheek pouch as an example of oral mucosa that is relatively easily manipulated [23]. Moreover, the lesions undergo a predictable progression through hyperplasia, mild and severe dysplasia before tumours begin to appear after approximately 12 weeks of application 3× per week. (Table 4) lists some autochthonous tumour models that have been employed in PDT studies.

**Table 4:** Autochthonous tumours Abbreviations: CBA, a strain of mice; DLI, drug light interval; FVB, another strain of mice; LED, light emitting diode; NMRI–HR-HR, another strain of mice; TPA, 12-O-Tetradecanoylphorbol-13-acetate.

Cancer type	Animal species	Carcinogen	PDT regimen	References
Oral cancer/dysplasia	Male Wistar rats	4-Nitroquinoline-1-oxide (4NQO)	Photofrin, DLI 24 h, 625 nm, 100 J/cm^2^, 60 mW/cm^2^.	[75]
Oral cancer/dysplasia	Male CBA mice	4-Nitroquinoline-1-oxide (4NQO)	ALA, DLI 5 h, 630 nm, 200 J/cm^2^, 125 mW/cm^2^	[76]
Oral cancer/dysplasia	Syrian Golden Hamster	DMBA	ALA, DLI 2.5 h, LED 638 nm, 275 J/cm^2^, 200 mW/cm^2^	[77]
Oral cancer/dysplasia	Syrian Golden Hamster	DMBA	Topical Photosan, DLI 16 min, 640 nm, 100 J/cm^2^, 320 mW/cm^2^, 313 s	(72)
Mammary tumours	Virgin Sprague–Dawley female rats	DMBA	Photogem® haematoporphyrin, DLI 24 h, LED 635 nm, 200 J/cm^2^,180 mW/cm^2^.	[78]
Skin tumour	FVB/N mice	DMBA/12-ο-tetradecanoylphorbol-13-acetate (TPA)	ALA, DLI 48 h, 635 nm, 120 J/cm^2^, 120 mW/cm^2^	[79]
Skin tumour	female hairless mice (NMRI–HR-HR)	UV irradiation-induced tumour	ALA-Me, DLI 4 h, 630–636 nm, 40 J/cm^2^, 20 mW/cm^2^	[80]

### Genetically-engineered mouse models of cancer

Genetically-engineered mouse models (GEMMs) of cancer have been constructed to more closely mimic the development of human disease in a predictable manner [24]. Since the 1980s, several types of GEMMs have been used, including transgenic, knockout and knockin mouse models [25]. Mutant mice that express oncogenes or dominant-negative tumour-suppressor genes governed by ectopic promoter and enhancer elements can be generated by pronuclear injection of cDNA constructs that contain promoter elements designed to restrict expression to certain tissues. GEMM can be produced by the direct injection of fertilized oocytes or by lentiviral transduction of embryonic stem cells. Targeted transgenesis provides an attractive alternative to random transgenesis by using integration mediated by recombination at specific sites allowing insertion of only a single copy of the transgene [26].

Many GEMM models of cancer have been developed including lung, colon, stomach, oesophagus, pancreas, liver, breast, ovary, prostate, bladder, kidney, brain and skin [25]. In some of these models, an additional application of a carcinogen needs to be used to actually initiate tumour development. Examples of these additional insults could be UV irradiation to initiate skin cancer or injection of dextran sulfate to initiate colon carcinogenesis. Despite the large number of GEMM, as yet only a few of these have been used to test PDT. These are listed in Table 5.

**Table 5:** GEMM Abbreviations: DLI, drug light interval; FVB, another strain of mice.

Tumour	GEMM	Inducible	PDT regimen	References
Breast	Male FVB/NTgN(WapHRAS)69LlnYSJL	No	Foscan-PEG, SC102; DLI 96 h, 652 nm, 40 J/cm^2^	[81]
Breast	FVB.Cg- Tg(WapHRAS)69Lln Chr Y ^SJL^/J		5-ALA for fluorescence detection of three different BrCa. DLI 75 min	[82]
	FVB/N-Tg(MMTV-PyVT)634Mul/J			
	C57BL/6J-Tg(WapTAg)1Knw			
Pancreas ductal adenocarcinoma	LSL-KRasG12D-p53-floxed-Pdx-1-Cre	No	Cathepsin-E cleavable ALA, DLI 1 h, 652 nm, 10 J/cm^2^, 50 mW/cm^2^, 3.5 min	[83]
Basal cell carcinoma	B6C3Fe-a/a-Ptchmes/+B6C3Fe-a/a-Ptch mes/+B6C3Fe-a/a-Ptch^mes^/+	UV exposure 20 weeks	MAL, DLI 3 h, 550 nm, 650 Hz, 7 J/cm^2^, 5 mW/cm^2^	[84]
Melanoma	MT-ret transgenic 304/B6	No	5-ALA, DLI 3 h, 630 nm, 200 J/cm^2^, 100 mW/cm^2^	[85]
Colon dysplasia	C57BL/6J-Apc(Min)	Dextran sulfate	Oral ALA for fluorescence detection of colon tumours, DLI 3 h	[86]

### *In vivo* imaging

In recent years, there has been an explosion of non-invasive imaging techniques applied to laboratory studies of cancer and anti-cancer therapy. Many of these *in vivo* imaging modalities are based on adaptation of those clinical modalities that are familiar to oncologists to be used in small animals. This classification includes such modalities as micro-positron emitting tomography (microPET) [27], micro-single photon micro-computed tomography (micro-SPECT) [28], micro-computed tomography (μCT) [29], high resolution ultrasound [30], photoacoustic tomography (PAT) [31]. However, another large group of small animal *in vivo* imaging modalities relies upon genetically-encoded reporter molecules and falls into the category of molecular imaging. The two most often used modalities in this class are (1) *in vivo* fluorescence imaging relying on engineered fluorescent protein reporters such as GFP; and (2) *in vivo* bioluminescent imaging relying on engineered luciferase reporters such as the firefly or marine luciferase enzymes.

### Infections

After cancer, infections represent the next most frequent application of PDT. The use of PDT against infections is motivated by the widespread and growing problem of antibiotic resistance, often called ‘the single biggest problem facing global health’ [32]. A very large number of studies have been published on the use of a wide variety of PS to kill or inactivate numerous classes of microorganisms including Gram-positive bacteria, Gram-negative bacteria, fungi, parasites and viruses. However, the number of studies on PDT for actual infections in animal models is much more limited (Table 6). Traditional methods for quantifying infection involve the killing of animals at various time-points, removal of the infected tissue, homogenization, serial dilution, plating on agar and colony counting. In recent years, the experimental study of PDT for infections has been facilitated by the use of *in vivo* bioluminescence imaging to non-invasively monitor the progression of the infection. Sensitive cameras can record non-invasively, in real time and with longitudinal monitoring, the anatomical location and growth of infectious microorganisms in living hosts [33]. Figure 5 shows an example of the use of this bioluminescence imaging technique to monitor PDT of a Gram-negative *Escherichia coli* infection mediated by pL-ce6 conjugate and red light, in excisional wounds on the back of BALB/c mice [34].

**Figure 5:**
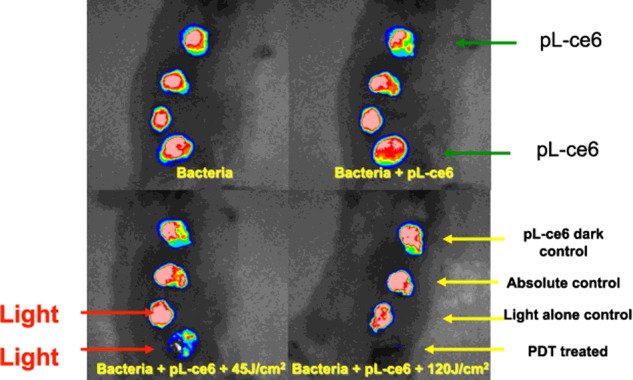
Set of images showing bioluminescence bacteria (*E. coli*) infecting incisional wounds on the back of BALB/c mice treated with PDT mediated by pL-ce6 and 660 nm light (**A**) Bioluminescent bacteria added to the wounds. (**B**) PS (pL-ce6 conjugate) is added to wounds 1 and 4. (**C**) Red (660 nm, 45 J/cm^2^) light is delivered to wounds 3 and 4 and a loss of bioluminescence is observed in wound 4. (**D**) At the completion of the light delivery (120 J/cm^2^), the bioluminescence in wound 4 (PDT) has been totally eliminated.

**Table 6:** PDT of infections in animal models Abbreviations: BCG, Bacillus Calmette-Guerin; BPD, benzoporphyrin derivative; DLI, drug light interval; IP, intraperitoneal; LED, light emitting diode; MB, methylene blue; MRSA, methicillin-resistant *Staphylococcus aureus*; PEI, polytheleneimine; TBO, toluidine blue O.

Animal model andapplication	Pathogenic microorganism	PDT Regimen	References
Subcutaneous injection of bacteria into dorsal skin of mice	*Pseudomonas aeruginosa*	Sn(IV) chlorin, DLI 15 min, 630 nm, 100 mW/cm^2^, 1600 s	[87]
Bacteria topically applied to burns in guinea pigs	MRSA methicillin resistant *Staphylococcus aureus*	Haemin/deuteroporphyrin, DLI 1 h, broad-band white, 8 mW/cm^2^, 10 min	[88]
Bacteria topically applied to excisional wounds on the backs of mice	Bioluminescent *E. coli*	Poly-L-lysine-chlorin(e6), DLI 0–30 min, 660 nm, 300 mW, 40 J/cm^2^, 50 mW/cm^2^	[89]
Topical application to excisional wounds on the backs of mice	Bioluminescent *P. aeruginosa*	Poly-L-lysine-chlorin(e6), DLI 30 min, 660 nm, 40 J/cm^2^, 100 mW/cm^2^	[90]
Bacteria injected into the muscles of mice	Bioluminescent *S. aureus*	pL-ce6, DLI 30 min, 665 nm, 160 J/cm^2^, 100 mW/cm^2^	[91]
Infected burns in mice	*S. aureus*	Cationic porphyrin Sylsens B, 635 nm, 423 J/cm^2^, 84 min	[92]
Infected burns in mice	*Acinetobacter baumannii*	PEI-ce6, DLI 15 min, 660 nm, 240 J/cm^2^, 100 mW/cm^2^	[93]
Oral candidiasis in nude mice	*C. albicans*	MB, DLI 10 min, 664 nm, 275 J/cm, 400 mW, 687.5 s	[94]
Leishmaniasis in hamsters	*Leishmania amazonensis*	MB, DLI 10 min, LED, 663 nm, 12 J/cm^2^, 5 mW/cm^2^, 1 h.	[95]
Subcutaneous granulomas of tuberculosis in male BALB/c mice	*Mycobacterium bovis* BCG	Verteporfin, BPD, DLI 1 h, 690 nm, 60 J/cm^2^ and 100 J/cm^2^	[96]
Otitis media with effusion (OME) in Mongolian gerbils	*Streptococcus pneumoniae* or *Hemophilus influenzae*	Haematoporphyrin, DLI 6 h, 632 nm, 90 J, 100 mW, 15 min	[97]
Periodontal disease in Wistar rats.	Natural Gram-negative anaerobic bacteria	TBO, DLI 1 min, 660 nm, 57.14 J/cm^2^	[98]
Osteomyelitis bacterial infection in Sprague–Dawley rats	Bioluminescent *S. aureus*	IP ALA, DLI 4 h, 635 nm, 75 J/cm2, 250 mW/cm^2^	[99]
Murine bacterial arthritis in knee	Bioluminescent methicillin-resistant *S. aureus* (MRSA)	Intra-articular MB, 660 nm, DLI 5 min, 160 J/cm^2^, 100 mW/cm^2^	[100]
Oral wound infections in Wistar rats	*Streptococcus spp*. and *Actinomyces viscosus*	TBO, 635 nm, DLI 10 min, 48 J/cm^2^, 246 mW, 2 min	[101]
Infection in excisional wounds of mice	*Vibrio vulnificus*	TBO, DLI 30 min, 560–780 nm, 200 J/cm^2^, 80 mW/cm^2^, 20 min	[102]

An interesting alternative animal model to study the pathogenesis and therapy of infections is the use of invertebrate hosts [35]. The invertebrate host that has been most frequently used is the larva of *Galleria mellonella*, the greater wax moth. The pathogens are injected into the larva, followed by injection of the PS and then by light delivery. This has been reported for *Enterococcus faecium* [36] and for *Candida albicans* [37] both of which were treated with methylene blue and red light.

### Miscellaneous indications

There has been a diverse range of diseases other than cancer and infections that have been treated with PDT in animal models. Some of these are listed in Table 7.

**Table 7:** PDT for other disease indications Abbreviations: BPD-MA, benzoporphyrin derivative monoacid ring A; DLI, drug light interval; MB, methylene blue.

Disease	Specialty	Animal model	PDT regimen	References
Choroidal neovascularization	Ophthalmology	Choriocapillary photo-occlusion in rabbits	Verteporfin BPD-MA, DLI 3 h, 692 nm, 10 J/cm^2^	[103]
Choroidal neovascularization	Ophthalmology	Argon-laser-induced injuries to choroid in monkeys	Verteporfin, liposomal BPD, DLI 50 min, 692 nm, 150 J/cm^2^, 600 mW/cm^2^	[104]
Atherosclerosis	Cardiology	Balloon-injured arteries in cholesterol fed rabbits	Etiopurpurin MV0611, DLI 24 h, 542 nm, 18 J/cm^2^, 90 s	[105]
Atherosclerosis	Cardiology	Balloon-injured arteries in cholesterol-fed Yucatan miniswine	Photofrin, DLI 24 h, 630 nm, 240 J/cm^2^, 1.28 mW/cm^2^, 188 s	[106]
Atrial fibrillation	Cardiology	Intravascular ablation to block cavotricuspid isthmus in dogs	Talaporfin sodium, continuous infusion, 663 nm, 10 W/cm^2^, 30 s	[107]
Atrial fibrillation	Cardiology	Atrioventricular block in rats	Talaporfin sodium, DLI 30 min, 670 nm, 10 J/cm^2^, 150 mW/cm^2^	[108]
Vascular wound healing	Vascular surgery	Balloon-injured carotid arteries in rats	Local MB, 660 nm, 100 J/cm^2^, 100mW/cm^2^	[109]
Wound healing	Dermatology	Excisional wounds in rats	ALA IP, DLI 24 h, 632 nm, 3 J/cm^2^, 5 mW	[110]
Hypertrophic scars	Dermatology	Excisional wounds in ears of rabbits	Topical ALA, DLI 3 h, 635 nm, 114.6 J/cm^2^, 20 min	[111]
Photoaging	Dermatology	UV irradiation of mouse skin 5 days/8 weeks	Topical ALA, DLI 4 h, 635 nm, 75 J/cm^2^	[112]

## CONCLUSIONS

It is almost a universally accepted axiom in cancer research that enormous numbers of mice and rats have been cured of cancer by a wide range of experimental therapeutics, but the translation into clinical practice has been disappointing more often than not. Nevertheless, the importance of designing and selecting the most appropriate animal models to investigate the ever-increasing range of new experimental therapeutic approaches has never been greater. This consideration applies even more to the rather complex techniques of PDT and aPDI (antimicrobial photodynamic inactivation), than it does to the more traditional testing of new pharmaceutical or biologic therapies. The multi-target nature of anti-cancer PDT (tumour cells, tumour vasculature and immune response) means that thought must be given to understanding the specific tumour biology of the model chosen. Moreover, since the light delivery is by definition a localized process, the anatomical location of orthotopic tumours becomes important.

When the testing of PDT in animal models of infectious disease is undertaken, some other points need to be considered. Firstly it is important to realize that human infections generally develop gradually from a relatively small initial infectious inoculum, rather than from a sudden large application of millions or tens of millions of CFU. Secondly, a high level of selectivity for microbial cells over host mammalian cells is needed, since the actual fraction by weight of microbial cells in even a serious infection is still very low. Thirdly, when the light is switched off, the generation of microbicidal ROS is halted and the microbial cells may be free to resume their growth unhindered.

The popularity of testing PDT for non-traditional indications involves designing animal models of non-cancer, non-infectious disease. A diverse assortment of conditions in cardiology, atherosclerosis, ophthalmology, neuroscience could in principle be treated with PDT.

Finally, for those laboratories that are not easily able to carry out studies on traditional mammalian animal models, there are new models described using invertebrate hosts and fertilized eggs. Large animal models have not been much studied in the PDT field, but as more new indications are being studied, studies on large animals can be expected to become more important.

## Abbreviations

ALA: aminolevulinic acid
CAM: chorioallantoic membrane
DMBA: 7,12-dimethyl-benz(a)anthracene
GEMM: Genetically-engineered mouse model
MHC: major histocompatibility complex
PDT: photodynamic therapy
PPIX: protoporphyrin IX
PS: photosensitizer
ROS: reactive oxygen species
SCID: severe combined immunodeficient
